# Gastroenteropancreatic Neuroendocrine Neoplasia Characterization in Portugal: Results from the NETs Study Group of the Portuguese Society of Endocrinology, Diabetes and Metabolism

**DOI:** 10.1155/2019/4518742

**Published:** 2019-08-01

**Authors:** A. P. Santos, J. Vinagre, P. Soares, I. Claro, A. C. Sanches, L. Gomes, I. Fernandes, A. L. Catarino, J. Preto, B. D. Pereira, A. P. Marques, F. Rodrigues, C. Amaral, G. Rocha, J. C. Mellidez, H. Simões, J. M. Lopes, M. J. Bugalho, On behalf of the NETs Study Group of the Portuguese Society of Endocrinology, Diabetes and Metabolism

**Affiliations:** ^1^Instituto Português de Oncologia do Porto, Francisco Gentil (IPOPFG), 4200-162 Porto, Portugal; ^2^Instituto de Investigação e Inovação em Saúde (i3S), 4200-135 Porto, Portugal; ^3^Instituto de Patologia e Imunologia Molecular da Universidade do Porto (IPATIMUP), 4200-465 Porto, Portugal; ^4^Faculdade de Medicina da Universidade do Porto (FMUP), 4200-319 Porto, Portugal; ^5^Centro Hospitalar de São João (CHSJ), 4200-319 Porto, Portugal; ^6^Centro Hospitalar de Lisboa Ocidental (CHLO), 1349-019 Lisboa, Portugal; ^7^Centro Hospitalar e Universitário de Coimbra (CHUC), 3000-075 Coimbra, Portugal; ^8^Centro Hospitalar Lisboa Norte, EPE (CHLN), 1649-035 Lisboa, Portugal; ^9^Centro Académico de Medicina de Lisboa (CAML), 1649-035 Lisboa, Portugal; ^10^Hospital da Luz, 1500-650 Lisboa, Portugal; ^11^Hospital Garcia de Orta, EPE, 2801-951 Almada, Portugal; ^12^Unidade Local de Saúde de Matosinhos, 4464-513 Senhora da Hora, Portugal; ^13^Instituto Português de Oncologia de Coimbra, Francisco Gentil (IPOCFG), 3000-075 Coimbra, Portugal; ^14^Centro Hospitalar do Porto-Hospital Santo António, 4099-001 Porto, Portugal; ^15^Centro Hospitalar Gaia/Espinho (CHGE), 4434-502 Vila Nova de Gaia, Portugal; ^16^Centro Hospitalar do Baixo Vouga (CHBV), 3810-501 Aveiro, Portugal; ^17^Portuguese Society of Endocrinology, Diabetes and Metabolism, Rua Fernando Vicente Mendes, 1B1600-892 Lisboa, Portugal

## Abstract

**Background:**

The incidence of gastroenteropancreatic neuroendocrine neoplasms (GEP-NENs) has been increasing in the last five decades, but there is no large-scale data regarding these tumours in Portugal. We conducted a cross-sectional, multicentric study in main Portuguese centers to evaluate the clinical, pathological, and therapeutic profile of GEP-NENs.

**Methods:**

From November, 2012, to July, 2014, data from 293 patients diagnosed with GEP-NENs from 15 centers in Portugal was collected and registered in an online electronic platform.

**Results:**

Median age at diagnosis was 56.5 (range: 15-87) years with a preponderance of females (54.6%). The most frequent primary sites were the pancreas (31.1%), jejunum-ileum (24.2%), stomach (13.7%), and rectum (8.5%). Data regarding hormonal status was not available in most patients (82.3%). Stratified by the tumour grade (WHO 2010 classification), we observed 64.0% of NET G1, 24.7% of NET G2, and 11.3% of NEC. Poorly differentiated tumours occurred mainly in older patients (*p* = 0.017), were larger (*p* < 0.001), and presented more vascular (*p* = 0.004) and lymphatic (*p* = 0.001) invasion. At the time of diagnosis, 44.4% of GEP-NENs presented metastatic disease. Surgery (79.6%) and somatostatin analogues (30.7%) were the most frequently used therapies of GEP-NENs with reported grading.

**Conclusion:**

In general, Portuguese patients with GEP-NENs presented similar characteristics to other populations described in the literature. This cross-sectional study represents the first step to establish a national database of GEP-NENs that may aid in understanding the clinical and epidemiological features of these tumours in Portugal.

## 1. Introduction

Neuroendocrine neoplasms (NENs) are a heterogeneous group of rare malignancies originating from endodermal cells with secretory capacity within the neuroendocrine system. Gastroenteropancreatic- (GEP-) NENs represent a subtype of these tumours, located either in the pancreas or in the gastrointestinal tract [1]. Although the incidence is low, it has been increasing significantly in the recent years; the age-adjusted incidence rate increased 6.4-fold from 1973 (1.09 per 100,000 persons) to 2012 (6.98 per 100,000 persons) [2]. Due to the long survival rate of patients with these tumours, the estimated 20-year limited-duration prevalence of NENs in the USA on January 1, 2014, was 171,321 [2]. The long survival reflects, besides the intrinsic biologic characteristics of neuroendocrine cells, the advances in diagnostic techniques and the awareness among clinicians [3].

NENs can be classified into functional and nonfunctional tumours according to the presence or absence of clinical symptoms associated with hormone overproduction [4]. Nonspecific symptoms are evident in the majority of nonfunctional cases resulting in a delay in diagnosis. NENs have been a subject of long debate regarding nomenclature, grading, and classification. The 2010 World Health Organization (WHO) classification, developed together with the European Neuroendocrine Tumour Society (ENETS), presented a significant progress by using two separate and complementary classification tools: histologic grading and site-specific staging system, classifying NENs according to the proliferation index (fraction of Ki-67 staining or number of mitotic counts) into grade 1 (G1), grade 2 (G2), and neuroendocrine carcinoma (NEC) [5]. In 2017, this WHO classification was updated, and the NENs are now divided into 3 main categories: mixed neuroendocrine-nonneuroendocrine neoplasms (MiNEN), NEN G1/G2/G3 (well-differentiated NEN), and NEC G3 (poorly differentiated NEN, large or small cell subtypes). The main differences in comparison with the 2010 classification are the Ki-67 index of NEN G1 tumours that was altered to less than 3% (instead of ≤2%) and an additional NEN G3 subcategory that was added to the well-differentiated NENs, with a labelling index of more than 20% for Ki-67 or more than 20 mitotic counts per 10 HPF. NEC G3 (poorly differentiated carcinomas) also require a Ki-67 proliferative index higher than 20%, as well as more than 20 mitotic counts per 10 HPF [6].

The aims of the available treatment options are to promote symptom relief, improve life quality, and ideally, a disease-free setting in patients which is largely dependent on primary tumour size and localization. These therapies vary from conservative procedures to pharmacologic and surgical management, and patterns of care differ between hospitals and countries depending on medical teams, experience and available resources.

Due to paucity of data on GEP-NENs in Portugal, the Neuroendocrine Tumours Study Group (GE-TNE) of the Portuguese Society of Endocrinology, Diabetes and Metabolism (SPEDM) sought to perform an observational study to present an outline of GEP-NEN patients followed up at the main Portuguese hospitals regarding their sociodemographic and clinical profiles (spectrum of symptoms at presentation, methods used in the diagnosis, and treatment modalities applied). These data will contribute towards the effort of developing a National Registry for effective monitoring of NENs and emphasize its importance as well as the need for multidisciplinary involvement for a comprehensive management of GEP-NENs in Portugal.

## 2. Materials and Methods

We designed a cross-sectional multicenter evaluation of patients diagnosed with GEP-NENs in 15 Portuguese centers that agreed to participate in the study. Inclusion criteria were patients with more than 18 years of age, a confirmed diagnosis of GEP-NEN based on histopathological, cytological, and/or biochemical/nuclear imaging findings, and a signed informed consent for study inclusion. Patients were consecutively enrolled in the study as they attended their medical appointment during a continuous 18-month period of the study. At the time of enrollment, data were collected directly from patients and from clinical files and submitted to an electronic platform. Variables included age, gender, GEP-NEN subtype, site of the primary tumour, WHO 2010 grading classification, tumour stage at diagnosis, symptoms at presentation, diagnostic procedures, hormonal and biochemical evaluations, treatment procedures, and duration of follow-up. Carcinoid syndrome was defined as values of 5-hydroxyindoleacetic acid (5-HIAA) equal or greater than twice the upper limit of the normal range plus flushing and/or diarrhea. Insulinoma diagnosis was based on hypoglycemic symptoms, Whipple triad, and/or a positive 72-hour prolonged fasting test. Gastrinoma diagnosis was based on clinical picture and gastrin levels greater than ten times the upper limit of the normal range, after excluding chronic atrophic gastritis and PPI (proton pump inhibitors) use. Imagiological procedures were evaluated according to primary tumour location. The tumour stage was classified as localized (confined to the organ of origin), regional (invasion of the surrounding organs or tissues or regional lymph nodes), or distant (spread to distant organs).

Ethical principles concerning ESP-GPP (Expanded Scope of Practice-Good Pharmacy Practicing), Helsinki Declaration, and National Legislation requirements were fulfilled.

Statistical analysis was performed with SPSS® statistics (software version 15.0). Categorical and continuous variables were summarized using descriptive statistics (frequencies for categorical variables and mean/standard deviation or median/interquartile range for continuous variables, as appropriate). Proportions were compared by the Chi-squared test or Fisher's exact test, as appropriate. Means were compared using the *t*-test or ANOVA.

## 3. Results

### 3.1. General Characteristics of the Population

A total of 314 cases were collected, whereas only 293 patients were included in the present study; the remaining 21 patients were excluded as they did not meet the inclusion criteria, such as lack of clinical information or the absence of informed consent. Data are summarized in Table 1.

Briefly, the cohort presented a 1 : 1.2 male to female ratio (133 males and 160 females), with a median age at diagnosis of 56.5 years (range: 15–87). The primary tumour site was predominantly the pancreas (31.1%), followed by the jejunum-ileum (24.2%), the stomach (13.7%), and the rectum (8.5%).

Clinically/hormonal functional syndrome was identified in 16.5% of patients: 17 presented criteria for carcinoid syndrome, 11 for insulinoma, and 4 for gastrinoma. No other hypersecreting tumours were detected in this series.

The majority of cases were diagnosed by histopathology or cytopathology, 86.7% and 5.8%, respectively, and less frequently (1.7%) by biochemistry, namely, in insulinomas.

According to the WHO 2010 classification, cases where graded as NET G1 (*n* = 158, 64.0%), NET G2 (*n* = 61, 24.7%), and NEC (*n* = 28, 11.3%); in 46 cases, data was not available. Information regarding extension of the disease was available in 214 cases and revealed localized disease in 35.5% of cases (including gastric, duodenum, and colorectal polyps) and distant disease in 44.4%. Regional spread was present in 20.1% of the cases.

The sociodemographic and clinical features of GEP-NEN patients, according to the tumour grade, are summarized in Table 2. NET G1 were more frequently detected in females (72.1%), whereas NET G2 and NEC were more common in males, 31.4% and 13.6%, respectively, (*p* = 0.020). There was a significant association between the WHO 2010 tumour grading and age at diagnosis (*p* = 0.017), with NEC being diagnosed at a median age of 62.5 years (range: 39–84) *vs.* 56.5 years (range: 32–80) for NET G2 and 54.7 years (range: 15–85) for NET G1. Patients with well-differentiated NENs presented a significantly higher mean body mass index (BMI) (*p* = 0.015) in comparison with NEC patients. There was a significant association of smoking and alcohol consumption with NET G2 (*p* = 0.007) and NEC (*p* = 0.037). NEC patients had less comorbidities than patients of the other two groups of NENs (57.6% *vs.* 71.4% in NET G1 and 75.8% in NET G2); these results were not statistically significant. There was a significant association between WHO 2010 tumour grading groups and primary tumour size at diagnosis, higher in NEC (*p* < 0.001). Vascular and lymphatic invasions were significantly more frequent in NEC (*p* = 0.004 and *p* = 0.001, respectively), whereas perineural invasion presented the same trend without statistical significance (*p* = 0.064).

Multiple endocrine neoplasia type 1 (MEN-1) syndrome was diagnosed in 4 patients; two patients had pancreatic tumours and two patients with gastric tumours. All patients with MEN-1 syndrome had primary hyperparathyroidism, and two patients had a pituitary adenoma and an adrenal adenoma, respectively.

### 3.2. Biochemical Tests

Biochemical data analysis concerning hormonal hypersecretion was informative in 32 patients (10.9%). Chromogranin A (CgA) equal or greater than twice the normal value was detected in 86 (51.2%) of the 165 patients evaluated (Table 3). Concerning specific markers, urinary 5-HIAA was evaluated in 115 patients and was positive in 47 (40.9%); of these, 17 patients presented carcinoid syndrome criteria. Insulinoma was identified in 11 patients (3.6%) either by Whipple's triad criteria and/or positive prolonged fasting test. Four sporadic gastrinomas were identified (Table 1).

### 3.3. Imaging Studies

The imaging modalities used as a diagnostic procedure—either for primary tumours or for metastases—are presented in Table 4. A computerized tomography (CT) scan was performed in 233 (79.5%) of the 293 patients and identified primary and/or metastatic tumour location in 79.5% of the evaluated cases. Octreoscan® was performed in 121 (41.3%) of the 293 patients and was informative in 63.6% of the evaluated cases. A ^68^Ga-positron emission tomography- (PET-) SSTR scan was used in 99 (33.8%) of the 293 patients and was informative in 75.8% of the evaluated cases. ^111^In-pentetreotide (^111^In-octreoscan®) (Octreoscan®) and ^68^Ga-PET-SSTR scan were mainly used in NET G1 and NET G2 patients, 89.8% and 93.1%, respectively. Fluorodeoxyglucose- (FDG-) PET was evaluated in 36 (12.3%) of 293 patients. Upper gastrointestinal endoscopy presented the highest efficiency in localizing oesophageal (3 out of 3, 100%), gastric (27 out of 30, 90%), and duodenal (17 out of 19, 89.5%) tumours. Echoendoscopy was valuable in the detection of duodenal (6 out of 6, 100%), pancreatic (25 out of 28, 89.3%), and gastric (7 out of 13, 53.8%) tumours. A colonoscopy was the main diagnostic procedure in colonic NEN detection (12 out of 12, 100%), as well as in rectal NENs (21/22, 95.5%). For midgut tumours, magnetic resonance imaging (MRI), CT, and video capsule were the mostly used imaging procedures; PET for somatostatin receptors (SSTR), ^68^Ga-PET-SSTR, demonstrated to be the most sensitive (94.1%) imaging tool.

### 3.4. Extension of the Disease

**Figure 1:**
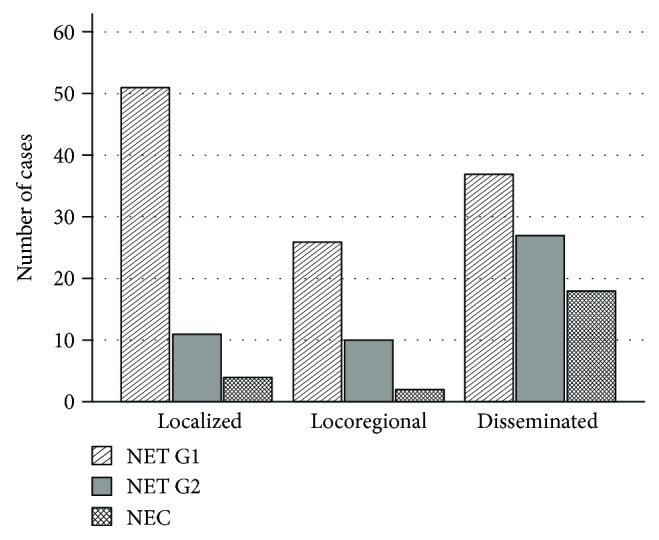
Extension of disease according to WHO 2010 classification.

Extension of the disease was evaluated in 186 patients (Figure 1 and Table 2). Localized disease was more frequent in NET G1 (44.7%). Regional disease was detected in 20.1% of the patients: 22.8% with NET G1, 20.8% with NET G2, and 8.3% with NEC. Metastases were present in 32.5% of patients with NET G1, in 56.3% with NET G2, and in 75.0% with NEC. Among cases with distant metastases at presentation (*n* = 82), 30.5% presented liver metastases. Bone metastases were detected in one patient with a NET G2 and two patients with NEC. Only one patient with NEC had lung metastases. Other sites of distant metastases included the peritoneum (five patients: one NET G1, one NET G2, and three NEC), adrenal glands (one patient with NEC), ovary (one patient with NET G1), and inferior vena cava (one patient with NET G1).

### 3.5. Treatment Procedures

Endoscopic removal of the tumours was possible in 40 patients with localized gastric, duodenal, and colorectal NENs. According to the WHO 2010 classification, either curative or cytoreductive surgery was performed in 125 out of 155 cases (80.6%) of NET G1, 48 out of 60 cases (80.0%) of NET G2, and 18 out of 25 cases (72.0%) of NEC (Table 5); overall, 191 of 240 patients (79.6%) were treated with surgery. Concerning patients with disseminated disease, 22 patients (18.2%) with NET G1, 9 patients (20.5%) with NET G2, and 8 patients (44.4%) with NEC were submitted to debulking surgery, mainly liver metastasectomy.

Although 95 patients presented liver metastases at diagnosis, locoregional ablative therapy, such as transarterial embolization (TAE), transarterial chemoembolization (TACE), radioembolization, or radiofrequency (RF)/thermoablation (TA), was only performed in 14 patients with well-differentiated NETs; 70.0% of the cases submitted to TAE and 75.0% submitted to RF/TA were NET G1. Only four patients were submitted to radioembolization, being three NET G1 and one NET G2.

**Figure 2:**
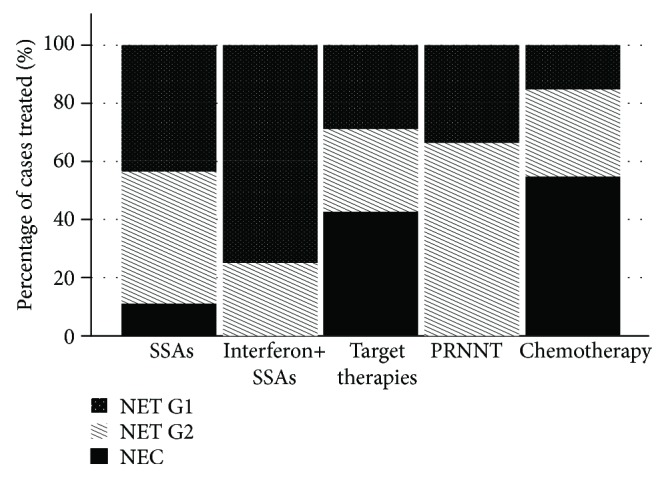
Cases submitted to different systemic therapies according to WHO 2010 classification.

Systemic therapy included somatostatin analogues (SSAs), interferon-*α*2b, target therapies with tyrosine kinase inhibitors and mTOR inhibitors, peptide receptor radiotherapy (PRRT), and chemotherapy (Table 5, Figure 2).

SSAs were mostly used in well-differentiated NETs (*p* < 0.001), comprising 20.4% of NET G1, 59.3% of NET G2, and 32.0% of NEC. Only 4 patients received combined treatment with SSAs and interferon-*α*2b. Target therapies as sunitinib and everolimus were used in seven (3.0%) patients: two with NET G1, two with NET G2, and three with NEC. Peptide receptor radiotherapy (PRRT) was used in nine (3.9%) of the patients, mainly well-differentiated NETs (33.3% NET G1 and 66.7% NET G2). Chemotherapy treatment was performed in 20 patients, mostly in NEC of the colon and the pancreas (11 patients; *p* < 0.001).

## 4. Discussion

GEP-NENs have been historically considered a rare and heterogeneous group of neoplasms. They comprise approximately 0.5% of all human cancers and 2% of gastrointestinal tumours [1]. New data from SEER 18 [2] reported a 6.5-fold increase in the annual incidence from 1973 to 2012 in NENs [2], reinforcing the need for research in this field. GEP-NENs often exhibit relatively indolent clinical courses and a delay in the diagnosis and tend to present metastases at the time of diagnosis, preserving the potential for lethal progression.

The present study was designed to characterize the overall scenario of GEP-NENs in Portugal, namely, the incidence and epidemiology of these tumours, sociodemographic and clinical profiles of the patients, and the patterns of care in a multicenter audit. Our results provide a comprehensive and relevant information on a group of neoplasm still poorly characterized, particularly, in Southern Europe. Published data from GEP-NEN in European countries is available in a French registration study [7], in a Spanish study of the Neuroendocrine Tumours Study Group Registry of Spain (RGETNE) [8], in an Italian epidemiological study [9], in a prospective Greek registry [10], and in the United Kingdom and Northern European countries [11–13]. Worldwide, the most characterized cohorts are from the United States of America (USA) [2, 14], and there is data available from Asian countries, such as China [15] and Japan [16].

Overall, our findings are in accordance with reports of NENs from other countries and corroborate that they are a heterogeneous group of tumours with a wide range of clinical presentation. We observed a similar gender ratio with a slight preponderance for females, as observed in a USA series [13, 14], Canadian series [17], and Italian study [9]. In our series, the pancreas was the most frequent primary tumour site, followed by the jejunum-ileum and the stomach. These findings are in agreement with data from Southern European countries, as the Italian and Greek cohorts [9, 10] as well as in China [18], but in contrast with other published studies [2, 7, 8, 17], where the gastrointestinal tract was reported as the most frequent primary site. These inconsistencies may be due to a referral bias and may suggest geographic and ethnic variation in the carcinogenesis of GEP-NENs. A recent publication stresses the differences in geographic and ethnic distribution, other than NEN fortuitous location and identification related to the current accuracy of the diagnostic methods [19], and points to the possibility of involved environmental risk factors. Prospective and larger studies will be useful to further clarify these findings.

The present study provides a comprehensive report on diagnostic and therapeutic procedures used in the current clinical practice in Portugal. Like the Spanish results reported by the RGETNE, in Portugal, there is a limited overall use of biochemical tests at diagnosis, namely, the general marker serum chromogranin A or urinary 5-HIAA quantification for midgut tumours.

In our cohort, as in another series [8], the most frequent functioning tumour was NEN with carcinoid syndrome, followed by insulinoma and apparently sporadic gastrinoma. No glucagonoma, VIPoma, somatostatinoma, or other rare syndromes were identified. It should also be taken into consideration that in 71.7% of the cases, the hormonal secretion by the tumour was not evaluated. This seems to reflect a low referral rate of patients to specialized centers, low participation of endocrinologists in the oncology team, and/or a limited laboratory support in some of the institutions that participated in this study. Our results highlight the ongoing demand for an adequate management of diagnostic, treatment, and follow-up work-out for patients with GEP-NENs. Most of the international epidemiological studies report data about localization, histological classification, and staging of GEP-NENs, but information about their hormonal secretion is sparse. Biochemical evaluation is important, not only for diagnostic purposes but also for therapeutic decision and monitoring of treatment responses, and an adequate assessment of tumour secretion is strongly encouraged. Genetic testing is also important when clinically indicated, as it allows for (1) a personalized life-long screening for prototypic tumours and their timely treatment, (2) the identification of affected family members that may benefit from this screening, and (3) appropriate genetic counseling. In our series, the majority of the cases lacked genetic evaluation for clinical suspicion of hereditary syndromes.

Histological classification of NENs is evolving as the WHO revised the nomenclature and classification of GEP-NENs in 2010 [5] and updated it in 2017 [6]. Histopathological characterization with immunohistochemistry markers such as chromogranin and synaptophysin is essential to make the diagnosis. The mitotic index and/or immunohistochemistry for Ki-67 labelling index is mandatory to generate the tumour grading [4]; these are minimum requirements for an accurate pathological classification. At the time of the inclusion of the patients in the present study, the histological classification was performed according to the 2010 WHO criteria, the up-to-date guidelines used for this study. Overall, in this study, the frequency of NET G1, NET G2, and NEC fits with other reports.

Tumour metastases at diagnosis represent an important prognostic marker [2]. In this series, distant metastases were detected in 44.4% of patient (NET G1: 32.5%; NET G2: 56.3%; and NEC: 75.0). This is consistent with other studies, as the Spanish and Italian studies [8, 9], where distant metastases were observed in 44% and 42% of patients, respectively, and contrasts with a lower rate of distant metastases at diagnosis in the Greek [10], Chinese [15], and Canadian [17] studies (25.0%, 6.0%, and 20.8%, respectively), as well as the SEER Registry (21.0%) [14]. An explanation for these differences may be due to the inclusion of cases from oncological institutions, where the proportion of metastatic disease is considerably higher. In this study, the oncological institutions, from Lisbon and Porto, contributed with 46% of the patients included.

Endoscopic therapy is the mainstay for types 1 and 2 gastric endocrine tumours and for localized duodenal and colorectal NENs. In this cohort, endoscopic therapy was performed mainly in those cases.

Surgery remains the treatment of choice for GEP-NENs, with curative intent whenever feasible. If the tumour is unresectable, several approaches are available to induce tumour debulking as a manner to control life-threatening symptoms due to hormone secretion and to increase patient survival and quality of life [20, 21]. In experienced centers, ablative therapies are a good option to treat liver metastatic disease [22]. Our results show that either primary or cytoreductive surgery was performed in the majority of the hospitals included and mainly in well-differentiated NENs. Ablative therapies were used in less than 5% of the patients probably due to the fact that few centers have this treatment available. This finding indicates the need of a referral of the patients to centers where they can benefit from these therapeutic options.

Currently, the standard of care for systemic treatment in advanced NET treatment is SSA, which proved to be effective in controlling excessive hormonal secretion [23, 24] and allowing long-term improvement in secretory symptoms in 30–70% of patients. Recent studies report an additional antiproliferative role of SSA in nonfunctioning midgut [25], pancreatic, and lung NENs [26], which reflected in the significant progression-free survival in the treated patients when compared with placebo. Other therapeutic options include biologic agents interfering with specific molecules of cell signaling pathways, *e.g.*, the mammalian target of rapamycin (mTOR) and vascular endothelial growth factor (VEGF), with everolimus and sunitinib, respectively, both approved for pancreatic NENs [27, 28]. Everolimus was also approved for the treatment of advanced nonfunctioning lung and gastrointestinal NENs [29]. Studies using oral chemotherapy with temozolomide and capecitabine are demonstrating promising results in well-differentiated pancreatic NENs [30]. However, classic cytotoxic drugs still continue to be the first-line therapy for poorly differentiated GEP-NENS and are effective (up to 60% response rates) in well-differentiated pancreatic NETs; however, early relapses often occur [31]. Concerning the therapeutic options in the present study, endoscopic therapies, either curative or cytoreductive surgery, and SSA treatment were the preferred options for the majority of patients. Somatostatin analogues (SSAs) were the most frequently used drugs in our study. Locoregional ablative therapy, PRRT, and target therapies were rarely used. Remarkably, PRRNT was more frequently chosen than target therapies. This fact was remarkable, as in the Portuguese National Health System, only one center offered this therapeutic modality at the time of the present study. As in other series and according to the guidelines, chemotherapy was the treatment of choice in NEC and was also an option in well-differentiated nonpancreatic NETs, which may reflect the inclusion of older cases and/or the absence of a referral to specialized centers.

The results obtained in this study represent the first comprehensive registry of GEP-NENs in Portugal performed by the Neuroendocrine Study Group of the Portuguese Society of Endocrinology, Diabetes and Metabolism. These provide a valuable insight into the epidemiology, current clinical practice, and therapy strategies of this heterogeneous disease and will set the ground for the development of a National Registry of NENs. These reinforce the need for a national clinical framework for GEP-NENs, in order to ensure a systematic surveillance of the disease and ultimately improve the diagnosis, clinical management, and outcome of NEN patients.

## Figures and Tables

**Table 1:** Patient general characteristics.

*Gender (n* = 293)	
Male, *n* (%)	133 (45.4)
Female, *n* (%)	160 (54.6)
*Age (years,n* = 293)	
Median (range)	59.9 (22-89)
*Age at diagnosis (years,n* = 291)	
Median (range)	56.5 (15^‡^-87)
*Race (n* = 293)	
Caucasian, *n* (%)	285 (97.3)
African, *n* (%)	1 (0.3)
Other or not specified, *n* (%)	7 (2.4)
*Type of diagnosis (n* = 293)	
Histopathological, *n* (%)	254 (86.7)
Cytological, *n* (%)	17 (5.8)
Biochemical, *n* (%)	5 (1.7)
Other or not specified, *n* (%)	17 (5.8)
*Primary tumour by localization (n* = 293)	
Pancreas, *n* (%)	91 (31.1)
Head, *n* (%)	28 (30.7)
Body, *n* (%)	29 (31.9)
Tail, *n* (%)	32 (35.2)
Not specified, *n* (%)	2 (2.2)
Jejunum-ileum, *n* (%)	71 (24.2)
Stomach, *n* (%)	40 (13.7)
Type 1, *n* (%)	23 (57.5)
Type 2, *n* (%)	9 (22.5)
Type 3, *n* (%)	7 (17.5)
Not specified, *n* (%)	1 (2.5)
Rectum, *n* (%)	25 (8.5)
Duodenum, *n* (%)	20 (6.8)
Appendix, *n* (%)	20 (6.8)
Colon, *n* (%)	16 (5.5)
Oesophagus, *n* (%)	3 (1.0)
Unknown primary tumour	7 (2.4)
*Tumour group by secretion*	
Carcinoid syndrome^∗^, *n* positive/total studied^∗∗^ (%)	17/115 (14.8)
Gastrinoma^$^, *n* positive/total studied^$$^ (%)	4/55 (7.3)
Insulinoma^&^, *n* positive/total studied^&&^ (%)	11/24 (45.8)
*Tumour group by grade (n* = 247*); WHO, 2010*	
NET G1, *n* (%)	158 (64.0)
NET G2, *n* (%)	61 (24.7)
NEC, *n* (%)	28 (11.3)
*Tumour group by stage (n* = 214*); TNM (ENETS)*	
Localized, *n* (%)	76 (35.5)
Locoregional, *n* (%)	43 (20.1)
Disseminated, *n* (%)	95 (44.4)

^‡^Patient was 15 y old at diagnosis, currently 22 y old at the time of the study; ^∗^carcinoid syndrome criteria: 5 − HIAA > 2 times the normal value and flushing and/or diarrhea; ^∗∗^cases with 5-HIAA quantification; ^$^gastrinoma criteria: gastrin ≥ 10 times the normal value and exclusion of types I and II gastric tumours; ^$$^cases with gastrin quantification; ^&^insulinoma criteria: hypoglycemic symptoms, Whipple triad, and/or positive 72-hour prolonged fasting test; ^$$^cases with insulin quantification.

**(a):** 

	NET G1	NET G2	NEC
Total no. of patients (*n* = 247)	158 (64.0)	61 (24.7)	28 (11.3)

**(b):** 

	NET G1	NET G2	NEC	*p*
Gender (*n* = 247)				
Male (*n* = 118), *n* (%)	65 (55.1)	37 (31.4)	16 (13.6)	**0.020**
Female (*n* = 129), *n* (%)	93 (72.1)	24 (18.6)	12 (9.3)	

Age (*n* = 247), years (mean (SD))	58.3 (12.8)	59.8 (12.7)	63.0 (12.9)	0.176
Age at diagnosis (*n* = 246), years (median range)	54.7 (15-85)	56.5 (32-80)	62.5 (39-84)	**0.017**

Weight (*n* = 190), kg (mean (SD))	71.8 (13.2)	76.9 (17.5)	68.7 (10.8)	**0.049**
BMI (*n* = 149), kg·m^−2^ (mean (SD))	27.0 (4.6)	28.6 (5.7)	24.6 (3.1)	**0.015**

Comorbidities (*n* = 231), *n* (%)	105 out of 147 (71.4)	44 out of 58 (75.8)	15 out of 26 (57.6)	0.233
Arterial hypertension (*n* = 235), *n* (%)	29 out of 150 (19.3)	5 out of 58 (8.6)	3 out of 27 (11.1)	0.139
Diabetes mellitus (*n* = 234), *n* (%)	17 out of 149 (11.4)	4 out of 58 (6.9)	1 out of 27 (3.7)	0.417
Dyslipidaemia (*n* = 239), *n* (%)	15 out of 154 (9.7)	3 out of 58 (5.1)	3 out of 27 (11.1)	0.508
Cardiovascular disease (*n* = 235), *n* (%)	8 out of 150 (5.3)	2 out of 58 (3.4)	1 out of 27 (3.7)	0.897
Family history of nonendocrine neoplasm (*n* = 167), *n* (%)	51 out of 105 (48.6)	22 out of 42 (52.4)	6 out of 20 (30.0)	0.254

Smoking (*n* = 173), *n* (%)	3 out of 110 (2.7)	4 out of 42 (9.5)	3 out of 21 (14.3)	**0.007**
Alcohol consumption (*n* = 163), *n* (%)	38 out of 106 (35.8)	22 out of 37 (59.5)	10 out of 20 (50.0)	**0.037**

Tumour dimension (*n* = 213), mm (mean (SD))	21.3 (19.9)	32.7 (23.5)	51.7 (34.9)	**<0.001**
Vascular invasion (*n* = 162), *n* (%)	34 out of 106 (32.1)	24 out of 41 (58.5)	9 out of 15 (60.0)	**0.004**
Lymphatic invasion (*n* = 155), *n* (%)	39 out of 103 (37.8)	25 out of 36 (69.4)	11 out of 16 (68.7)	**0.001**
Perineural invasion (*n* = 119), *n* (%)	26 out of 84 (31.0)	9 out of 25 (36.0)	7 out of 10 (70.0)	0.064

**(c):** 

	NET G1	NET G2
*Hormonal status*		
Functioning (*n* = 32)^a^	17 out of 32 (53.1)	6 out of 32 (18.6)
Carcinoid (*n* = 17)^b^	8 out of 17 (47.0)	5 out of 17 (29.4)
Gastrinoma (*n* = 4)^c^	2 out of 4 (50.0)	1 out of 4 (25.0)
Insulinoma (*n* = 11)^d^	7 out of 11 (63.6)	0
Nonfunctioning, (*n* = 20)^e^	12 out of 20 (60.0)	5 out of 20 (25.0)

**(d):** 

	NET G1	NET G2	NEC	*p*
MEN-1 syndrome (*n* = 213)^§^	2 out of 137 (1.5)	2 out of 51 (3.9)	0 out of 25 (0.0)	0.575

Stage (*n* = 186)				
Localized, *n* (%)	51 out of 114 (44.7)	11 out of 48 (22.9)	4 out of 24 (16.7)	**0.001**
Locoregional, *n* (%)	26 out of 114 (22.8)	10 out of 48 (20.8)	2 out of 24 (8.3)	
Disseminated, *n* (%)	37 out of 114 (32.5)	27 out of 48 (56.3)	18 out of 24 (75.0)	

Cases missing WHO tumour classification grading: ^a^*n* = 9, ^b^*n* = 4, ^c^*n* = 1, ^d^*n* = 4, and ^e^*n* = 3. ^**§**^Cases reported as not presenting MEN-1 syndrome clinical features (no genetic testing was performed for unsuspicious cases).

**Table 3:** Biochemical tests.

Biochemical tests	Positive results, *n* (%)
Chromogranin A (*n* = 168)	86 (51.2)
5-HIAA (*n* = 115)	47 (40.9)
Insulin (*n* = 25)	11 (44.0)
Gastrin (*n* = 55)	25 (45.5)
Glucagon (*n* = 8)	0
VIP (*n* = 9)	0
ACTH (*n* = 17)	0
GH (*n* = 12)	0

VIP: vasoactive intestinal peptide; ACTH: adrenal corticotrophin; GH: growth hormone.

**Table 4:** Imaging modalities used for the diagnostic procedure either for primary sites and metastasis.

	Oesophagus	Gastric	Pancreas	Appendiceal	Duodenum	Jejunum-ileum	Colon	Rectum	UPT^∗^	Positive/total exams
Upper endoscopy	3/3 (100.0)	27/30 (90.0)	—	—	17/19 (89.5)	—	—	—	1/4 (25.0)	48/56 (85.7)
Echoendoscopy	—	7/13 (53.8)	25/28 (89.3)	—	6/6 (100.0)	—	—	10/10 (100.0)	—	48/57 (84.2)
Video capsule	—	—	—	—	—	8/9 (88.9)	—	—	—	8/9 (88.9)
Double balloon	—	—	—	—	—	1/1 (100.0)	—	—	—	1/1 (100.0)
Colonoscopy	—	—	—	—	—	12/33 (36.4)	12/12 (100.0)	21/22 (95.5)	—	45/67 (67.2)
Entero-CT	—	—	—	—	—	4/4 (100.0)	—	—	—	4/4 (100.0)
Entero-MRI	—	—	—	—	—	11/11 (100.0)	1/1 (100.0)	—	—	12/12 (100.0)
US scan	—	5/12 (41.7)	33/41 (80.5)	1/4 (25.0)	2/2 (100.0)	23/27 (85.2)	1/2 (50.0)	0/3 (0.0)	1/1 (100.0)	66/92 (71.7)
CT scan	3/3 (100)	10/22 (45.5)	71/77 (92.2)	4/11 (36.4)	13/17 (76.5)	52/62 (83.9)	13/15 (86.7)	9/20 (45.0)	6/6 (100.0)	181/233 (77.7)
MRI	—	0/3 (0.0)	38/44 (86.4)	1/1 (100.0)	5/5 (100.0)	11/13 (84.6)	2/4 (50.0)	4/9 (44.4)	2/2 (100.0)	63/81 (77.8)
^111^In-pentetreotide^‡^	—	6/17 (35.3)	26/36 (72.2)	2/6 (33.3)	8/12 (66.7)	25/30 (83.3)	5/8 (62.5)	2/9 (22.2)	3/3 (100.0)	77/121 (63.6)
^68^Ga-PET-SRP	—	5/12 (41.7)	26/31 (83.9)	2/5 (40%)	1/2 (50.0)	32/34 (94.1)	2/2 (100.0)	5/11 (45.5)	2/2 (100.0)	75/99 (75.8)
PET-FDG	2/2 (100.0)	0/5 (0.0)	10/17 (58.8)	0/1 (0.0)	1/1 (100.0)	2/4 (50.0)	3/5 (60.0)	—	1/1 (100)	19/36 (52.8)

^‡^Octreoscan®; ^∗^UPT: unknown primary tumour; CT: computed tomography; MRI: magnetic resonance imaging; PET-FDG: positron emission tomography-(^18^F) fluorodeoxyglucose.

**(a):** 

	Gastric, *n* (%)	Duodenum, *n* (%)	Rectum, *n* (%)
Endoscopic therapy (*n* = 40)	21 (52.5)	4 (10.0)	15 (37.5)

**(b):** 

	NET G1	NET G2	NEC	*p*
Surgical therapy (*n* = 240)^$^	125 out of 155 (80.6)	48 out of 60 (80.0)	18 out of 25 (72.0)	0.607
Surgery of metastases (*n* = 183)^$^	22 out of 121 (18.2)	9 out of 44 (20.5)	8 out of 18 (44.4)	0.055

*Liver ablative therapy*				
TAE (*n* = 199)^$^	7 out of 131 (5.3)	3 out of 49 (6.1)	0 out of 19 (0.0)	0.781
RFA (*n* = 101)^$^	3 out of 61 (4.9)	1 out of 27 (3.7)	0 out of 13 (0.0)	>0.999

*Systemic therapies*				
Somatostatin analogues (*n* = 231)^$^	31 out of 152 (20.4)	32 out of 54 (59.3)	8 out of 25 (32.0)	**<0.001**
Interferon (*n* = 231)^$^+SSAs	3 out of 152 (2.0)	1 out of 55 (1.8)	0 out of 24 (0.0)	>0.999
Target therapies^∗^ (*n* = 231)^$^	2 out of 153 (1.3)	2 out of 53 (3.8)	3 out of 25 (12.0)	**0.020**
PRRNT^∗∗^ (*n* = 230)^$^	3 out of 150 (2.0)	6 out of 55 (10.9)	0 out of 25 (0.0)	**0.021**
Chemotherapy (*n* = 244)^$^	3 out of 157 (1.9)	6 out of 60 (10.0)	11 out of 27 (40.7)	**<0.001**

^$^Number of cases with information. TAE = transhepatic arterial embolization; RFA = radiofrequency ablation; PRRNT: peptide receptor radionuclide therapy. ^∗^Sunitinib; ^∗∗^^177^Lu-THERA.

## Data Availability

The clinical data used to support the findings of this study are included within the article.
