# Erratum to “Gastroenteropancreatic Neuroendocrine Neoplasia Characterization in Portugal: Results from the NETs Study Group of the Portuguese Society of Endocrinology, Diabetes and Metabolism”

**DOI:** 10.1155/2020/9184324

**Published:** 2020-06-18

**Authors:** A. P. Santos, J. Vinagre, P. Soares, I. Claro, A. C. Sanches, L. Gomes, I. Fernandes, A. L. Catarino, J. Preto, B. D. Pereira, A. P. Marques, F. Rodrigues, C. Amaral, G. Rocha, J. C. Mellidez, H. Simões, J. M. Lopes, M. J. Bugalho, On behalf of the NETs Study Group of the Portuguese Society of Endocrinology, Diabetes and Metabolism

**Affiliations:** ^1^Instituto Português de Oncologia do Porto, Francisco Gentil (IPOPFG), 4200-072 Porto, Portugal; ^2^Instituto de Investigação e Inovação em Saúde (i3S), 4200-135 Porto, Portugal; ^3^Instituto de Patologia e Imunologia Molecular da Universidade do Porto (IPATIMUP), 4200-465 Porto, Portugal; ^4^Faculdade de Medicina da Universidade do Porto (FMUP), 4200-319 Porto, Portugal; ^5^Centro Hospitalar de São João (CHSJ), 4200-319 Porto, Portugal; ^6^Instituto Português de Oncologia de Lisboa, Francisco Gentil (IPOLFG), 1099-023 Lisboa, Portugal; ^7^Centro Hospitalar e Universitário de Coimbra (CHUC), 3000-075 Coimbra, Portugal; ^8^Centro Hospitalar Lisboa Norte, EPE (CHLN), 1649-035 Lisboa, Portugal; ^9^Centro Académico de Medicina de Lisboa (CAML), 1649-035 Lisboa, Portugal; ^10^Hospital da Luz, 1500-650 Lisboa, Portugal; ^11^Hospital Garcia de Orta, EPE, 2801-951 Almada, Portugal; ^12^Unidade Local de Saúde de Matosinhos, 4464-513 Senhora da Hora, Portugal; ^13^Instituto Português de Oncologia de Coimbra, Francisco Gentil (IPOCFG), 3000-075 Coimbra, Portugal; ^14^Centro Hospitalar do Porto-Hospital Santo António, 4099-001 Porto, Portugal; ^15^Centro Hospitalar Gaia/Espinho (CHGE), 4434-502 Vila Nova de Gaia, Portugal; ^16^Centro Hospitalar do Baixo Vouga (CHBV), 3810-501 Aveiro, Portugal; ^17^Centro Hospitalar de Lisboa Ocidental (CHLO), 1349-019 Lisboa, Portugal; ^18^Portuguese Society of Endocrinology, Diabetes and Metabolism, Rua Fernando Vicente Mendes, 1B1600-892 Lisboa, Portugal

In the article titled “Gastroenteropancreatic Neuroendocrine Neoplasia Characterization in Portugal: Results from the NETs Study Group of the Portuguese Society of Endocrinology, Diabetes and Metabolism” [1], the affiliation for I. Claro was labeled incorrectly. The correct affiliation of the author I. Claro is Instituto Português de Oncologia de Lisboa, Francisco Gentil (IPOLFG), 1099-023 Lisboa, Portugal.
